# Pathway Analysis of GWAS Provides New Insights into Genetic Susceptibility to 3 Inflammatory Diseases

**DOI:** 10.1371/journal.pone.0008068

**Published:** 2009-11-30

**Authors:** Hariklia Eleftherohorinou, Victoria Wright, Clive Hoggart, Anna-Liisa Hartikainen, Marjo-Riitta Jarvelin, David Balding, Lachlan Coin, Michael Levin

**Affiliations:** 1 Division of Medicine, Department of Paediatrics, Imperial College London, London, United Kingdom; 2 Division of Epidemiology, Department of Epidemiology and Public Health, Public Health and Primary Care, Imperial College London, London, United Kingdom; 3 Department of Clinical Sciences/Obstetrics and Gynecology, University of Oulu, Oulu, Finland; 4 Institute of Health Sciences and Biocenter Oulu, University of Oulu, Oulu, Finland; 5 Department of Child and Adolescent Health, National Institute of Health and Welfare, University of Oulu, Oulu, Finland; Peninsula Medical School, United Kingdom

## Abstract

Although the introduction of genome-wide association studies (GWAS) have greatly increased the number of genes associated with common diseases, only a small proportion of the predicted genetic contribution has so far been elucidated. Studying the cumulative variation of polymorphisms in multiple genes acting in functional pathways may provide a complementary approach to the more common single SNP association approach in understanding genetic determinants of common disease. We developed a novel pathway-based method to assess the combined contribution of multiple genetic variants acting within canonical biological pathways and applied it to data from 14,000 UK individuals with 7 common diseases. We tested inflammatory pathways for association with Crohn's disease (CD), rheumatoid arthritis (RA) and type 1 diabetes (T1D) with 4 non-inflammatory diseases as controls. Using a variable selection algorithm, we identified variants responsible for the pathway association and evaluated their use for disease prediction using a 10 fold cross-validation framework in order to calculate out-of-sample area under the Receiver Operating Curve (AUC). The generalisability of these predictive models was tested on an independent birth cohort from Northern Finland. Multiple canonical inflammatory pathways showed highly significant associations (p 10^−3^–10^−20^) with CD, T1D and RA. Variable selection identified on average a set of 205 SNPs (149 genes) for T1D, 350 SNPs (189 genes) for RA and 493 SNPs (277 genes) for CD. The pattern of polymorphisms at these SNPS were found to be highly predictive of T1D (91% AUC) and RA (85% AUC), and weakly predictive of CD (60% AUC). The predictive ability of the T1D model (without any parameter refitting) had good predictive ability (79% AUC) in the Finnish cohort. Our analysis suggests that genetic contribution to common inflammatory diseases operates through multiple genes interacting in functional pathways.

## Introduction

The technological development of high throughput genotyping has provided a powerful tool to examine the genetic basis of disease through Genome-Wide Association Studies (GWAS). These studies have considerably increased the number of known genes associated with common diseases [1]. However, given the large number of markers typed and the stringent statistical criteria necessary to minimize false positive hits [2], so far only the most significant associations have been established. Attempts to increase the power of GWAS to detect genes with moderate effects by increasing sample size through meta-analysis may be less effective in detecting rarer variants, and is limited by inter-population heterogeneity. It is likely that the genetic associations reported to date represent only the tip of the iceberg of genes contributing to disease risk, and that the majority of genes still remain hidden within the statistical “noise” inherent in this approach [3]. As a result, much of the genetic information which may emerge from GWAS remains unutilised and the question of how many genes contribute to disease susceptibility, how they interact to cause disease, and the extent to which disease pathogenesis might be genetically predicted remains largely unknown [4].

Disease susceptibility is likely to depend on the cumulative effect of variants in multiple genes interacting in functional pathways. We use the term “interacting” in the biological sense to define genes whose products act within functional pathways, to alter the function or expression of other components of a pathway leading to a biological output. This “pathway” interaction is distinct from the statistical use of the term to define epistatic interaction, which is defined in the context of a particular phenotype and can be tested by looking at the correlation structure of mutations conditional on a phenotypic outcome (case vs. control for example).

**Figure 1:**
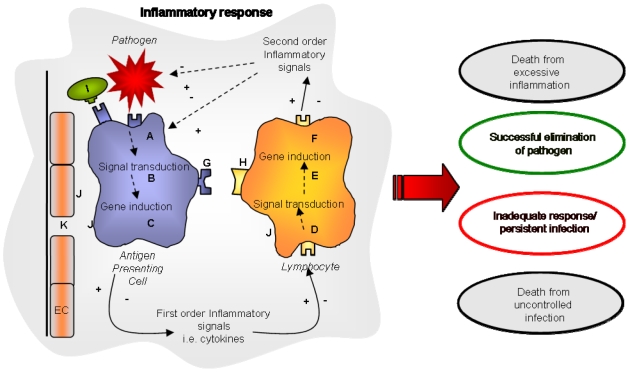
Inflammatory response to a pathogen. Pathogen recognised by pattern recognition receptors on phagocytic cell (A) or plasma opsonins (I). Signal induction (B) and first order inflammatory genes (C) are induced leading to release of inflammatory signals. These bind to receptors (D), leading to activation of signal transduction pathways and gene induction of second order inflammatory mediators (E, F). These act as effectors of the inflammatory response (Red Arrow) or as positive or negative regulators. Inflammation upregulates cell adhesion molecules (J) and those involved in transendothelial migration (K). Genetic variants (A–J) will interact to alter the intensity and nature of the response, and may determine different outcomes. Individuals making an excessive inflammatory response may succumb to overwhelming inflammation, while those making an inadequate response may fail to clear the pathogen. EC = endothelial cell.

If we consider the genetic regulation of the immune response, multiple genes contribute to the response to any pathogen - some acting as positive and others as negative regulators [5] (Figure 1). The pattern of gene variants within inflammatory pathways will determine the intensity and nature of an individual's immune response to pathogens and thus the outcome of different infectious diseases encountered throughout life [6], [7].

The same gene variants which result in rapid activation of a vigorous inflammatory response to infection may have the disadvantage of increasing the risk of auto-immune and inflammatory diseases later in life [8], [9]. Pro-inflammatory mediators such as TNF, IL-12, IL-1, IL-6 and IFN-γ, essential for containment of microbial pathogens [10], [11], are also associated with inflammatory processes seen in common auto-immune diseases such as RA [12], CD [13], or T1D [14]. Conversely, treatments that reduce inflammation, such as anti-TNF therapy for CD or RA [15] are associated with increased risk of opportunistic infection [16], suggesting that pathways involved in inflammatory diseases are also involved in resistance to infectious diseases. We postulated that the genetic contribution to common inflammatory diseases would be determined by multiple gene variants in the same inflammatory pathways involved in host response to infectious diseases (Figure 1).

We show here that the application of a pathway approach to GWAS yields new insight into the biology of CD, T1D and RA pathogenesis by implicating novel biological pathways as well as identifying new gene associations in known pathways. We also show that multiple SNP variants in these pathways can be used to build predictive models of disease risk, thus providing a new picture of how multiple gene variants combine to contribute to disease risk.

## Results

### Shared and unique disease pathways for each disease

We used the novel statistical approach (described in detail in the methods section) to evaluate the combined effect of multiple genetic variants in the genes comprising canonical immune and inflammatory pathways.

Highly significant associations were observed between key inflammatory pathways and the three inflammatory diseases (Table 1). Jak-STAT signalling, antigen processing and presentation, T cell activation pathway, cell adhesion molecules, hematopoietic cell lineage and NK cell mediated cytotoxicity showed high levels of significance in all three autoimmune diseases (*P*<10^−4^ to *P*<10^−20^). However certain pathways showed evidence for association with one disease; the pathways of NOD2 (*P*<10^−4^ to *P*<10^−15^), IL-23 and TNF (*P*<10^−20^), IL-12 and TGF-β (*P*<10^−6^), TLR2 signalling (*P*<10^−5^ to 10^−9^), classical MAPK (*P*<10^−10^) and B cell activation (*P*<10^−8^) with CD; TLR3 (*P*<10^−4^), T-cell activation via PLC and via NFAT (*P*<10^−4^) and ABC transporters (*P*<10^−16^) with T1D; TLR9 signalling via IRF5 (*P*<10^−6^) and purine metabolism (*P*<10^−5^) with RA (Table 1).

**Table 1:** 

PATHWAY	CD	T1D	RA	HT	BD	CAD	T2D
***Pattern recognition receptors***							
TLR2^1^	***3.5E-05***	0.029	0.041	0.257	0.187	0.386	0.357
TLR2/1^1^	***9.1E-06***	0.030	0.026	0.197	0.185	0.318	0.367
TLR2/6^1^	***2.4E-05***	0.020	0.065	0.129	0.129	0.179	0.316
TLR3^1^	0.001	**3.3E-04**	0.037	0.319	0.198	0.246	0.145
TLR4 (MyD88 dependent)^1^	0.020	0.010	0.040	0.199	0.102	0.469	0.335
TLR4 (MyD88 independent)^1^	0.018	0.018	0.029	0.152	0.124	0.343	0.262
TLR5/TLR7/TLR8/TLR9^1 2^	0.127	0.062	0.397	0.380	0.154	0.342	0.119
TLR2-IRF5	**2.7E-06**	0.225	0.001	0.116	0.085	0.389	0.477
TLR2/1-IRF5	**3.1E-09**	0.228	0.001	0.129	0.066	0.453	0.442
TLR2/6-IRF5	**8.1E-05**	0.171	0.001	0.179	0.066	0.102	0.330
TLR4 (MyD88 dependent)-IRF5	0.022	0.076	0.017	0.173	0.078	0.555	0.482
TLR5/TLR7/TLR8-IRF5^2^	0.441	0.804	0.032	0.315	0.013	0.394	0.004
TLR9-IRF5	0.002	0.058	**1.6E-06**	0.220	0.112	0.638	0.361
***Signal transduction***							
Jak-STAT signalling	***2.5E-07***	***1.9E-12***	***4.4E-09***	0.005	0.011	0.166	0.045
MAPK: All	***7.7E-09***	***1.5E-06***	0.119	0.005	0.054	0.020	0.003
MAPK: Classical	***1.8E-10***	0.001	0.164	0.009	0.121	0.033	0.001
MAPK: JNK & p38	***2.2E-06***	0.027	0.139	0.053	0.158	0.046	0.030
NFKB	0.030	0.016	0.010	0.842	0.533	0.333	0.294
NOD1^1^	0.097	0.324	0.633	0.003	0.141	0.001	0.489
NOD2 (via GRIM19)^1^	***4.4E-04***	0.055	0.237	0.184	0.102	0.205	0.210
NOD2 (via RICK)^1^	**1.3E-15**	0.587	0.528	0.005	0.151	0.045	0.287
***Second order cytokines***							
IL-1^1^	0.003	0.217	0.127	0.101	0.157	0.516	0.496
IL-6	***3.5E-04***	***4.2E-04***	0.064	0.140	0.826	0.221	0.426
IL-10	***8.4E-06***	***2.1E-04***	0.083	0.378	0.683	0.116	0.836
IL-12	***4.9E-06***	4.32E-03	0.003	0.324	0.949	0.523	0.747
IL-18	0.017	0.062	0.032	0.220	0.391	0.287	0.287
IL-23	***0.0E+00***	0.017	0.063	0.012	0.709	0.637	0.301
TNF^1^	***0.0E+00***	0.017	0.026	0.045	0.244	0.635	0.283
TGF-β	**2.6E-05**	0.004	0.035	0.102	0.502	0.155	0.218
***Antigen processing and presentation***							
All^1^	***1.4E-04***	***0.0E+00***	***0.0E+00***	0.012	0.016	0.256	0.577
MHC I	0.073	***0.0E+00***	***0.0E+00***	0.291	0.266	0.420	0.521
MHC II	***1.9E-05***	***0.0E+00***	***0.0E+00***	0.021	0.043	0.810	0.545
***B–cell activation***							
All^1^	***5.2E-08***	0.005	0.656	0.005	0.026	0.196	0.006
AKT^1^	0.039	0.550	0.223	0.015	0.021	0.059	0.004
AP1	***7.6E-11***	0.037	0.578	0.146	0.136	0.403	0.004
NFAT	***1.7E-04***	0.002	0.702	0.010	0.009	0.221	0.067
PKC^1^	0.037	0.050	0.553	0.245	***1.1E-04***	0.323	0.135
***T–cell activation***							
All^1^	***2.5E-11***	***1.3E-07***	***2.4E-05***	***4.1E-05***	0.004	0.069	0.002
AP1	***5.3E-07***	0.002	0.112	0.124	0.293	0.516	0.003
NFAT	0.004	***1.0E-04***	0.025	0.021	0.004	0.452	0.047
PLC^1^	0.002	***1.3E-04***	0.009	0.028	0.153	0.480	0.007
ICOS/CD28^1^	***4.1E-05***	0.227	0.029	0.001	0.060	0.024	0.352
Cytokines/receptors	***4.3E-04***	***6.0E-06***	***2.5E-06***	0.156	0.005	0.055	0.123
Cytokines/receptors/Jak-STAT/suppressors	***2.4E-04***	***1.2E-09***	***1.1E-04***	0.007	0.011	0.023	0.014
IFN-γ	**1.7E-05**	***8.6E-06***	0.018	0.065	0.089	0.186	0.630
***Signalling molecules and interaction***							
Cell adhesion molecules: All	***0.0E+00***	***0.0E+00***	***0.0E+00***	0.081	***3.1E-04***	***4.6E-04***	***2.0E-04***
APC: T cell	***5.0E-06***	***0.0E+00***	***0.0E+00***	0.211	0.001	0.618	0.184
Tc cell: target cell	0.010	***0.0E+00***	***0.0E+00***	0.577	0.284	0.561	0.438
Th cell: B cell	***2.5E-04***	***0.0E+00***	***0.0E+00***	0.068	0.035	0.363	0.554
Leukocyte: platelet	0.208	0.868	0.631	0.208	***3.5E-05***	0.680	0.139
Leukocyte: endothelial cell	0.029	0.095	0.257	0.194	***2.2E-04***	0.190	0.452
Neural cells	***7.6E-05***	0.123	0.125	0.191	0.045	0.001	0.001
Cytokine-cytokine receptor interactions	**4.2E-15**	***2.7E-12***	0.002	0.042	0.026	0.194	0.104
***Others***							
ABC transporters	0.021	***2.2E-16***	0.004	0.350	0.003	0.276	0.166
Cell communication	0.014	***8.3E-05***	0.007	0.064	***1.6E-04***	0.051	0.009
Complement: All	0.080	0.323	0.051	0.245	0.551	0.484	0.206
Haematopoietic cell lineage: All	***6.3E-10***	***0.0E+00***	***0.0E+00***	0.006	***2.8E-04***	0.019	0.070
Leucocyte transendothelial migration	0.013	0.575	0.102	0.418	0.003	0.059	0.203
Natural killer cell mediated cytotoxicity	***3.3E-10***	***0.0E+00***	***1.4E-09***	0.003	0.001	0.100	0.431
Neutrophil activation	***4.5E-05***	0.002	0.043	0.061	0.290	0.043	0.605
Purine metabolism	1.04E-02	0.026	***3.2E-05***	0.174	0.474	0.010	0.367
Pyrimidine metabolism	7.02E-02	0.160	0.039	0.389	0.377	0.590	0.280
Type 1 diabetes pathway from KEGG	***4.8E-07***	***0.0E+00***	***0.0E+00***	0.231	0.045	0.672	0.940
***Non-inflammatory pathways***							
Urea cycle	0.928	0.081	0.771	0.318	0.153	0.257	0.069
Citrate Cycle (TCA cycle)	0.829	0.935	0.331	0.197	0.235	0.761	0.327
Arachidonic Acid metabolism	0.411	0.844	0.289	0.157	0.806	0.338	0.192

1 Addition of NFKB did not change the result.

2 Although shown together, these were considered as separate pathways.

3 Additional pathways analyzed are shown in Table S3 & S4.

Almost all of the pathways under investigation showed no evidence of association with the non-inflammatory diseases (Table 1). However, some signal of association was detected in pathways with previously identified or biologically plausible link to a non-inflammatory disease. For instance, association between the B cell signalling via protein kinase C (PKC) pathway and bipolar disorder is consistent with reports that PKC activity has a role in pathophysiology of bipolar disease [17].

No association was seen for metabolic pathways that are not expected to have a biological link to the inflammatory diseases (Table 1 and web-based additional material Table 20).

### Common and unique key gene variants for each disease

To identify the genes and SNPs predominantly responsible for the pathway effect, we applied variable selection and model fitting on all the SNPs within associated pathways, within the framework of 10-fold CV. The models developed during CV consisted on average of 205 SNPs (149 genes) in T1D, 350 SNPs (189 genes) in RA and 493 SNPs (277 genes) in CD (Table S1). For all, except the smallest pathways, the signal for pathway association arises from the cumulative effect of many gene variants (Figure S1). Furthermore the significance of the majority of pathways was not dependent on the established significant (single SNP trend test *P*<5×10^−7^) hits, as when the pathway statistic was repeated after excluding significant hits, and known associations, the pathway statistic remained significant for the majority of associated pathways (Table S2).

**Figure 2:**
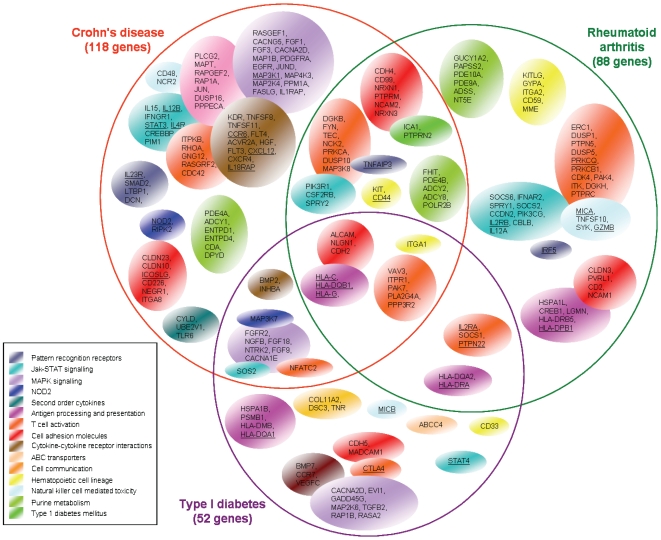
Genes identified by variable selection in all 10 folds of cross-validation for T1D, RA, CD. Key genes that showed consistent association with each disease had at least one mapping SNP selected in all 10 logistic models of CV. Genes are grouped in their pathways, which are shown as bubbles. Pathways are colour-coded in agreement with Table 1 and Supplementary Tables. Overlapping bubbles represent pathways that share key genes. Underlined genes correspond to associations that have been reported in previous association studies.

We reasoned that the genes selected in all ten CV models represent a “core” set of genes showing consistent association with the disease. This set comprised a total of 52 genes for T1D, 88 genes in RA and 118 genes in CD as shown in Figure 2 (SNPs shown in web-based additional material Tables 11–13). Only 12 genes were common to all three diseases, 11 were shared by CD and T1D, 5 shared by T1D and RA, and 26 were shared by RA and CD. The majority of identified genes were unique to each disease. Common genes included the major histocompatibility complex HLA-DQB1, HLA-G and HLA-C from the antigen processing and presentation pathway; PPP3R2, PLA2G4A, ITPR1, VAV3 and PAK7 from the T cell activation pathways; and the cell adhesion molecules ALCAM, NLGN1, ITGA1 and the cadherin CDH2.

Several of the genes we identified have been associated with autoimmune diseases in previous GWAS [18]–[22]. Of note is that our analysis identified IL12B, ICOSLG, STAT3, CCR6 for CD which have only been identified in subsequent meta-analysis of three separate GW scans [22], as well as IL18RAP which was identified in a recent case-control study [23]. For T1D, STAT4 was associated with the disease in follow-up candidate gene studies [24], [25] as was PRKCQ [26]–[28] (Figure 2). The previously identified genes found by our approach include for T1D, genes in the HLA region, CTLA4, PTPN22 and IL-2RA. For RA; PRKCQ and GZMB (these showed nominal significance *P*<10^−4^ to 10^−5^ in the WTCCC study), IL2RA, IL2RB and TNFAIP3 (these showed a modest evidence of association *P*<10^−5^ to 5×10^−7^ in WTCCC study), and MICA [29]–[31] as well as IRF5 [32], [33] that was confirmed by meta-analysis [34]. Similarly for CD, the well established associations with NOD2, IL23R and the recently implicated IL12B and STAT3 were all confirmed. However, in addition to these established associations we also identified other components of key pathways contributing to disease, such as RIPK2 and MAP3K7 from the NOD2 pathway in CD. We also identified genes in novel pathways: in T1D, a number of genes controlling T cell activation were selected including the calcium channel ITPR1, the calcium dependent phospholipase PLA2G4A, the regulatory subunit of calcineurin PPP3R2 and the calcineurin dependent transcription factor NFATC2 suggesting a role for calcineurin/nuclear factor of activated T-cells (NFAT) signalling in susceptibility of type 1 diabetes.

TNF plays a critical role in inflammation in RA and CD [12], [13] and has been a major target for therapeutic antibody treatments. The TNF pathway was significant in CD only, and key components selected include two enzymes that are regulators of NF-κB signalling–a negative regulator of NF-κB, the deubiquitinating enzyme CYLD [35] (rs7342715) and TNFAIP3 (rs7753394) both a deubiquitinating enzyme and a ubiquitin ligase [36]. Both of these enzymes deubiquitinate NEMO and, when knocked out *in vivo*, lead to inflammatory bowel disease [37]. Genetic variants near TNFAIP3 have recently been associated with RA (see below) as well as ulcerative colitis and CD [18], [38] and was associated in our study for both CD (rs7753394) and RA (rs6920220) (see below).

Considering the possible involvement of TLR signalling in RA, we found that only TLR9 signalling via IRF5 was significant among TLR pathways. TLR9 is constitutively expressed on B cells that are critical in the pathogenesis of rheumatoid arthritis [39]. The plausibility of this pathway is strengthened by the finding that several genes downstream of TLR9 were also selected; IRF5 (rs3807306) and the negative regulators SOCS1 (rs11074956, rs243325) and TNFAIP3 (rs6920220). It is of interest that recent studies show an association of the IRF5 gene with RA [32], [33], and 2 studies link region 6q23, flanked by TNFAIP3 and OLIG3 with RA susceptibility [40], [41].

### Genomic prediction of disease risk

**Figure 3:**
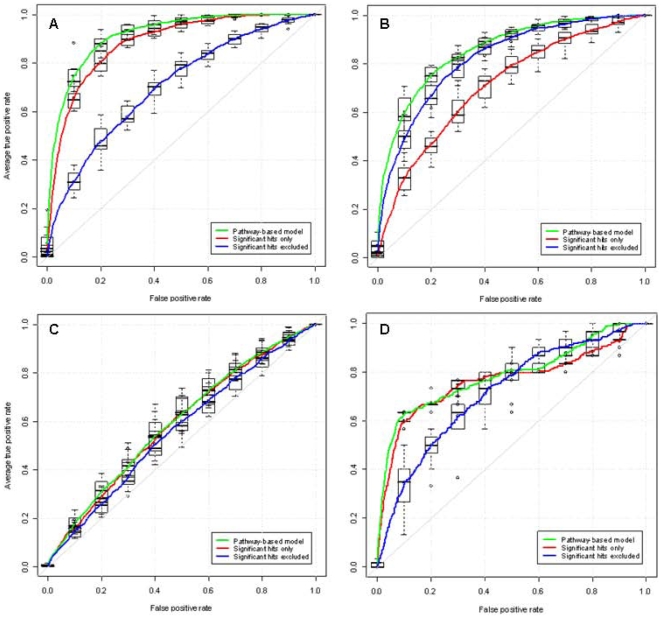
ROC curves showing the average predictive performance for T1D, RA and CD. True positive rate and false positive rate for predicting case/control status for A) type 1 diabetes, B) rheumatoid arthritis, C) Crohn's disease on the WTCCC dataset and D) ROC showing the average predictive performance of the T1D models built on the UK WTCCC dataset and applied to the 4,763 subjects in the Northern Finland 1966 Birth Cohort. Each colored line is the average ROC of the 10 models fitted during CV. The green curves show the performance of the models, as built by the variable selection algorithm. Blue curves show the performance of the same models with all significant hits (individual trend test *P*<5×10^−7^) and SNPs in LD (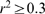) removed. Red curves show the predictive performance of the models formed only by the previously excluded SNPs (significant hits and SNPs in LD). In T1D (A) the area under the average ROC curves is 91%, 71% and 84%, in RA (B) it is 85%, 81%, 70% and in CD (C) 60%, 56%, 58% for the pathway-derived models (green-curves), the pathway-derived models excluding the significant hits (blue curves) and the significant-hit models (red-curves) respectively. In (D) the AUC of the green, blue and red ROC is 0.79, 0.71 and 0.76 respectively.

We next investigated how well the combination of gene variants selected by variable selection can predict disease in individuals. We used ten fold cross-validation (see methods) to build predictive models on 90% of the cases and controls, which are then tested on the remaining 10%. The process was then repeated using a different 90% and 10% of the cohorts on each occasion. The sensitivity and specificity of the models for each disease are shown in ROC curves (Figure 3A–C). The area under the average ROC (AUC) is 91%, 85% and 60% for T1D, RA and CD respectively, which correlates inversely to the number of SNPs in the models for each disease.

The predictive models, as expected, contained some of the well-established markers associated with each disease. To evaluate the extent to which prediction was driven by these significant hits, we split each model into two models without any refitting of the coefficients; a model excluding all SNPs with single-SNP *P*<5×10^−7^ as well as any SNPs in LD with these (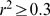); specifically for T1D and RA we also removed any SNPs that mapped to the MHC gene clusters in chromosome 6 [42] and a model with only these excluded SNPs. As shown in Figure 3, a large proportion of the predictive power comes from established associations (Figure 3A–C red curves, T1D 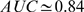, RA 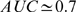, CD 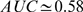), however the SNPs identified by the pathway analysis have predictive power in the absence of these hits (Figure 3A–C blue curves, T1D 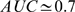, RA 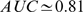, CD 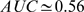) and also increase the predictive power when added to the significant hits (Figure 3A–C green curves, T1D 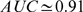, RA 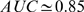, CD AUC 0.60). In all three diseases, the pathway-derived models had greater predictive value than the significant hits alone. Remarkably for RA, the SNPs identified by the pathway approach, excluding the significant hits, have higher predictive value than the significant hits on their own.

### Validation Study

In order to test the generalisability of our approach to other populations, and to investigate its applicability in the general population (rather than a case-control design), we tested the T1D predictive models in the Northern Finnish Birth cohort (NFBC). Each of the 10 T1D models trained on different 90% subsets of the WTCCC samples were used to calculate disease risk, without any parameter refitting. As the NFBC was typed on a different platform to the WTCCC, those SNPs which were not in common between the two genotyping platforms were imputed (see methods). The 10 T1D models trained on 90% of the WTCCC predicted on average over 60% of the Finnish T1D cases with a false positive rate of 10%, compared to 73% in the original UK sample (Figure 3D, green curve 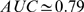). The single model trained on the entire WTCCC T1D case-control dataset achieved almost the same predictive power (Figure S2, green curve, 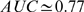). Although the significant SNPs (*P*<5×10^−7^) accounted for a considerable proportion of the predictive power (Figure 3D and Figure S2, red curves, 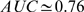), the additional SNPs identified by our approach contribute to the predictive power (Figure 3D and Figure S2, green curves) and have predictive value on their own (Figure 3D and Figure S2, blue curves, 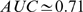).

### Visualisation of genomic risk

We reasoned that each SNP has a different effect on disease predisposition either increasing or decreasing the risk due to its functional effect on the regulation of the overall pathway output (Figure 1). We categorized SNPs as *adverse* or *protective* on the basis of their coefficient in the CV model (relative to the minor allele for additive effects). As shown in Figure S3, each SNP exerts an *adverse* or *protective* effect through dominant, recessive, additive or heterozygous modes. In T1D for example, there are two *adverse* HLA-DQB1 variants (rs9273363/rs9275418) and two *protective* HLA-DQA1 variants (rs9272723/rs9270986), and PTPN22 (rs6679677) is adverse for both T1D and RA. Figure S3 also illustrates the differing magnitude of effect of individual SNPs, with many showing only a small effect, but less common variants having more powerful *adverse* or *protective* effects.

**Figure 4:**
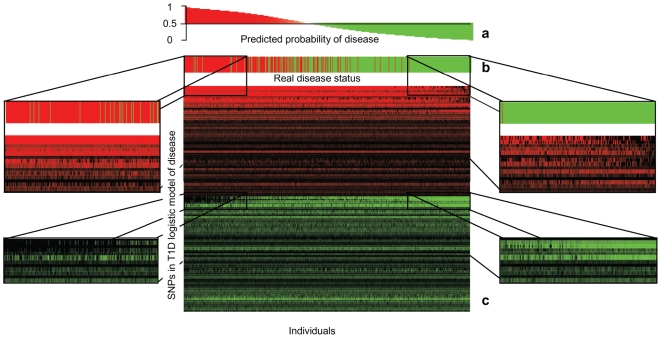
Variant SNPs carried by cases with type 1 diabetes and controls. (a) Predicted probability of being a case, (b) Actual case or control status. Patients shown in red and controls in green. The model correctly assigns the majority of cases at the extreme left, and controls at extreme right, with less predictive ability in the middle. (c) Individual patients or controls are displayed in columns and each row represents one SNP. Red indicates an *adverse* and green a *protective* SNP. Intensity of colour indicates disease log-odds from the predictive model. The magnified sections show regions where very marked differences between cases and controls can be readily seen.

In order to display for any individual, the pattern of rare and common variants, and the *protective* or *adverse* effect of each variant, in Figure 4 we plot for every individual (columns) their genotype at each SNP. We used red to indicate an *adverse* and green a *protective* SNP (rows). The intensity of colour reflects the effect of the SNP in the model (Methods S1). This provides a way of visualising the predictive models. Each individual seems to carry a unique combination of *adverse* and *protective* variants, which may represent a personal “genomic fingerprint” of disease predisposition. Despite the vast number of different combinations, a pattern is revealed where patients (left side) compared to healthy individuals (right side) are seen to carry a higher number of *adverse* genotypes and fewer number of *protective* genotypes and vice versa.

## Discussion

Our pathway analysis has shown that variation in conserved canonical inflammatory pathways underlies genetic susceptibility to T1D, CD, and RA. Although a number of pathways we have identified contain genes implicated in susceptibility to these diseases in previous single SNP based studies, our analysis presents a new perspective on the number of contributing pathways, the number of genes within pathways that interact to determine disease occurrence, and also identifies novel pathways and genes associated with each disease. Furthermore our analysis provides new insight into the pathways that are common as well as those unique to the three diseases, and suggests that genetic influence operates through functionally interacting genes.

Pathway-based approaches have been employed in previous studies [43]–[48], but have focused less on the underlying mechanisms that affect disease occurrence. Our approach not only enables the pathways associated with disease to be identified, but provides a method to identify the individual genes and their SNPs within the pathway that are predominantly responsible for the genetic effect. Our approach relies on the existence of well-defined biological pathways involved in inflammation, and our hypothesis that the cumulative effect of mutations in these pathways are likely to affect disease susceptibility in T1D, CD and RA. In many other diseases the relevant pathways are not yet as clearly identified. However, as the understanding of the function of genes increases [49], [50], the same approach may be readily applied to other diseases.

The number of genes associated through GWAS with CD, T1D and RA has been increasing progressively through the use of meta-analysis. In order to place the findings of our pathway-based approach in the context of what has been found using conventional single SNP analysis in the original WTCCC study, in previous studies or in more recent meta-analysis, we have tabulated in web-based additional material Tables 17–19, the previously reported associations for CD, RA, and T1D and related these to the genes implicated in our analysis. These tables show that several genes, which were not significant in the initial WTCCC analysis, but were identified by our approach, have now been confirmed in subsequent meta-analysis or candidate gene studies. One obvious concern of the pathway approach is that it can only evaluate the genetic contribution of genes known to act within pathways. As shown in web-based additional material Tables 17–19 several of the previously reported associations are genes not present in our inflammatory pathways, and thus are “missed” by our approach. However, there are many other genes which are identified by our analysis that have also been identified by meta-analysis or in large studies subsequent to WTCCC studies and which were “missed” by the WTCCC single SNP analysis. These genes suggest that the pathway approach may be a useful method for identification of real associations without recourse to meta-analysis, and using smaller cohorts than required for some of the confirmed associations. In web-based additional material Tables 17–19 we also show that there are genes that are implicated by our pathway-based approach which have not been found in previous studies, and thus require validation in independent data sets. While these associations have yet to be replicated, their predictive performance during cross-validation and the validation of the gene set identified on the WTCCC in predicting T1D in the NFBC suggests that these genes are plausible associations. As data from other studies on CD and RA become available, these novel associations can be validated either in GWAS or in candidate gene studies.

We have identified gene/SNP sets within inflammatory pathways which appear to have predictive value for disease occurrence. We stress that our evaluation of these gene sets as predictive markers of disease status was not aimed at assessing their clinical use in disease prediction. Instead we aimed to use the predictive performance of the models as an indication of the importance of genetic influence on disease occurrence [46], [51].

The validation of our T1D models on a different population provides the most stringent test of our approach. The predictive models trained on the UK WTCCC cohort predicted development of T1D in over 60% of the Finnish patients who developed disease by age 30 (with a false positive rate of 10%). Although the level of prediction was lower than that on the WTCCC cohort (true positive rate = 73%, false positive rate = 10%), which may suggest that the initial predictive power on the WTCCC was over-inflated, the lower performance may also be due to biological or technical factors, such as the use of imputed SNPs in the Finnish samples, and because the Finnish population differs from UK Caucasians in haplotypic structure [52]. However, the predictive power was still higher than achieved in other studies [53]–[55]. Definitive assessment of the predictive performance of models developed on one cohort (such as the WTCCC) will require validation studies to be undertaken on a second cohort from the same population and ethnic groups, and with direct genotyping undertaken on the same SNPs.

Although much of the predictive power in T1D was due to the MHC complex effects and other known associations, the remaining SNPs in our models have substantial predictive value in their own right, and remarkably in RA, the genes not meeting GWAS significance provide better predictive value than the significant hits alone. The lower predictive performance of the models in CD may reflect that this disease (which has a number of well recognised clinical phenotypic subgroups [51]) is more heterogeneous in its genetic origins, a possibility also suggested by the higher number of SNPs identified by variable selection in CD.

The strategy of using curated biological pathways has some limitations. As noted previously [56], this approach is dependent on the quality and completeness of the curated biological pathways used as input. Another obvious concern is that it can only evaluate the genetic contribution of genes known to act within pathways. Genes which are not yet within the accepted “canonical” pathways used for the analysis will be missed by our approach. For example some of the genes which were associated with T1D in the recent meta-analysis [26], were not included in the current curated pathways (e.g. PTPN2, C1QTNF) included as a starting point for our analysis. However, biological understanding of the function of genes in pathways is evolving rapidly, and currently available lists of “canonical” pathways will inevitably expand rapidly in the future as new data on function of genes becomes available. The strength of the single SNP approaches which have been used in most previous GWAS analysis is that they are “hypothesis free” and can bring to light associations with genes not previously suspected as having roles in a particular disease. Our pathway approach may be useful to complement the findings of significant associations at the single SNP level. Once a gene has been implicated by association at the single SNP level, all the other genes in the pathway in which the associated gene functions can then be included in the analysis, to identify other genes which may be acting in concert to produce the overall genetic effect.

Defining whether a particular SNP is part of, or regulates a gene can also be problematic, and can result in functionally important trans-regulatory SNPs being missed and will also miss the effect of SNPs in gene deserts. Large pathways or pathways with genes that reside in large LD blocks are likely to inflate the pathway statistic, although we accounted partially for this potential bias via the permutations procedure. These limitations however can each be addressed as functional pathways become better defined and understanding of gene function improves, through linking genomic and gene expression and/or proteomic data.

Our approach suggests a new picture of how variation in multiple genes linked in functional pathways contributes to inflammatory disease susceptibility and provides a useful tool to reveal the hidden information of GWAS that would be missed in single SNP analysis. We suggest that a biological pathway-based approach is likely to be valuable in elucidating the genomic mechanisms underlying common diseases and may identify new pathways as therapeutic targets.

## Methods

The flow chart in Figure S4 outlines the sequential steps in our analysis, and more detailed statistical methods are described in the supplementary section (Methods S1 and S2 and Figure S5).

### Patient Cohorts

The study was approved by the Clinical Research Governance Office of Imperial College London (Reference ICREC_9_1_11). All data were analyzed anonymously. We analysed the raw anonymous genotypic data from the Wellcome Trust Case Control Consortium (WTCCC) study on 14,000 Caucasian UK patients and 3000 controls genotyped on the Affymetrix 500K mapping array. The cohort included 7 common diseases; Crohn's disease, rheumatoid arthritis, type 1 diabetes, hypertension, type 2 diabetes, bipolar disorder and coronary artery disease and has been described in detail [18]. For the validation study we used anonymous data from 4,763 individuals in the Northern Finland 1966 Birth Cohort (NFBC 1966) [57] genotyped on the Illumina Infinium 370cnvDuo array, thirty of whom were ultimately diagnosed as having T1D.

### Pathway selection

We selected key canonical pathways associated with the innate and acquired immune response to pathogens (Figure 1 and Table S3 and S4), defined using the KEGG [58] and Ingenuity Pathways Analysis 6 databases, supplemented from the literature. We examined 84 pathways containing 1415 genes and 20,309 SNPs within 10KB of the genes. As negative controls, we selected metabolic pathways which biologically were not expected to contribute to inflammatory disease susceptibility (Table S3 and web-based additional material Table 9). Genes in all examined pathways are shown in the web-based additional material located at http://www1.imperial.ac.uk/medicine/people/l.coin/. A summary of the numbers of genes and SNPs used for each stage of analysis is shown in Table S1.

### Test for pathway association: cumulative trend test statistic

To evaluate the overall genetic contribution of a given pathway, we developed a cumulative trend test statistic 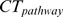 by summing the Armitage trend test statistic over all of the SNPs in the pathway. We estimated the parameters of a parametric approximation of the null distribution of the statistic by fitting a skew normal distribution to results obtained from 1000 random permutations of case/control labels. This procedure was carried out separately for each disease and given pathway. Pathway significance was defined at 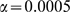, i.e. significance level 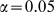 Bonferroni corrected for ∼100 pathways.

### Variable selection and logistic regression

To identify the genes (and SNPs) predominantly responsible for the pathway effect, we collected the SNPs within pathways with 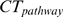 *P*<0,005 and applied HyperLasso [59] a variable selection algorithm designed to build a predictive model of disease risk. HyperLasso [59] fits a logistic regression model while performing variable selection to generate models with relatively few predictors. Variable selection and model fitting were performed under the framework of 10-fold cross validation (CV).

### Model evaluation and ROCs

To evaluate the performance of the predictive logistic models, we displayed the average sensitivity/specificity across all 10 trials via Receiver Operating Characteristic (ROC) plots and calculated the area under the ROC curves (AUC) [60].

### Validation in an independent cohort

The validation of our predictive models on an independent dataset was carried out in two ways; to achieve direct comparability between the level of prediction on the WTCCC data and the NFBC 1966 data, we used the same 10 models trained during cross-validation on a 90% subset of the WTCCC dataset on the independent dataset; we also fitted a single model on the entire WTCCC data and tested its ability to predict T1D in the subjects of the NFBC.

## Supporting Information

Figure S1 Fraction of the associated pathways selected on average by variable selection. The bar charts show for each disease the average number of genes in a pathway, selected during variable selection, divided by the total number of genes in that pathway and expressed as a percentage. Only associated pathways are shown. The total number of genes in a pathway are shown in parenthesis after the pathway name. The colour-coded bars for each disease are not stacked (i.e. they are not summated).
(0.85 MB TIF)

Figure S2 Single model fitted on the entire WTCCC T1D cases and controls when applied to the Northern Finland 1966 Birth Cohort. The area under the ROC curve for the pathway-derived model is 0.77 (green curve), for the same model but with all significant hits (single SNP trend test P<5×10^−7^) and the SNPs in LD (r^2^≥0.3) excluded is 0.69 (blue curve) and for the model with only the excluded SNPs is 0.74 (red curve).
(0.43 MB TIF)

Figure S3 The allelic architecture of the logistic regression models of disease risk. Minor allele frequency vs. beta coefficient for each SNP retained in the fitted logistic regression model from first round of cross-validation. Additive, recessive, dominant and heterozygous effects are displayed by black squares, blue dots, mauve triangles and cyan diamonds respectively. Adverse vs. protective SNPs have a positive vs. negative beta value respectively. Labels are given for all SNPs with beta greater than 0.25. In T1D there are a number of SNPs with large adverse effects acting in a dominant and additive manner and hence a significantly different sum of additive (t-test, P = 2×10e-06) and dominant (P = 2×10e-06) effects between cases and controls. In RA, we observe SNPs with large additive adverse and protective effects resulting in a significant difference in the sum of additive (P = 2×10e-14), as well as dominant (P = 6×10e-04) and recessive (P = 0.014) effects. In CD there are fewer SNPs with large effect yet still significant differences for additive (P = 4e-03) and heterozygous (P = 0.04) cumulative effects.
(1.17 MB TIF)

Figure S4 Stepwise procedure for the pathway-based analysis.
(0.66 MB TIF)

Figure S5 Histogram, density function and q-q plots of various distribution fits to permutation data of the cumulative trend test statistic. The plot on the top left corner shows the histogram and the fitted skew normal density function of the cumulative trend test statistic calculated from 10,000 permutations of cases/control label for the IL-1 pathway in CD and the top middle plot shows the QQ-plot of the fitted skew normal distribution. The next four plots correspond to QQ-plots of four distribution fits to the same null distribution. The P-value of Kolmogorov-Smirnof goodness of fit test statistic is depicted in the legend of each plot.
(0.41 MB TIF)

Table S1 Summary statistics at each stage of the analysis.
(0.02 MB XLS)

Table S2 P-values of the pathway statistic over the examined pathways without any SNPs with single-marker P<5×10^−7^. P-values in bold and scientific format correspond to pathways that preserved their statistical significance even after the removal of the significant hits. P-values in italics stand for pathways that were statistically significant with the significant hits included but not without.
(0.02 MB XLS)

Table S3 List of examined pathways with the corresponding numbers of genes and SNPs. The column of significant hits shows the number of SNPs with individual trend test P<5×10^−7^ within each pathway. Bold highlighting denotes association of a pathway with the disease. The last column shows the table index in the web-based additional material which contains lists of genes for every pathway.
(0.02 MB XLS)

Table S4 Additional inflammatory pathways analyzed. These pathways are not shown in Table 1, either because they showed no association to any disease (e.g. pyrin) or showed association but were combined into one to be used for downstream analysis (e.g. haematopoietic cell lineage).
(0.03 MB XLS)

Methods S1 (11.21 MB PDF)
