# Supplementary figures and images for: Pathway Analysis of GWAS Provides New Insights into Genetic Susceptibility to 3 Inflammatory Diseases
Source: PLoS One. 2009 Nov 30;4(11):e8068. doi: 10.1371/journal.pone.0008068 (PMC2778995; doi:10.1371/journal.pone.0008068)

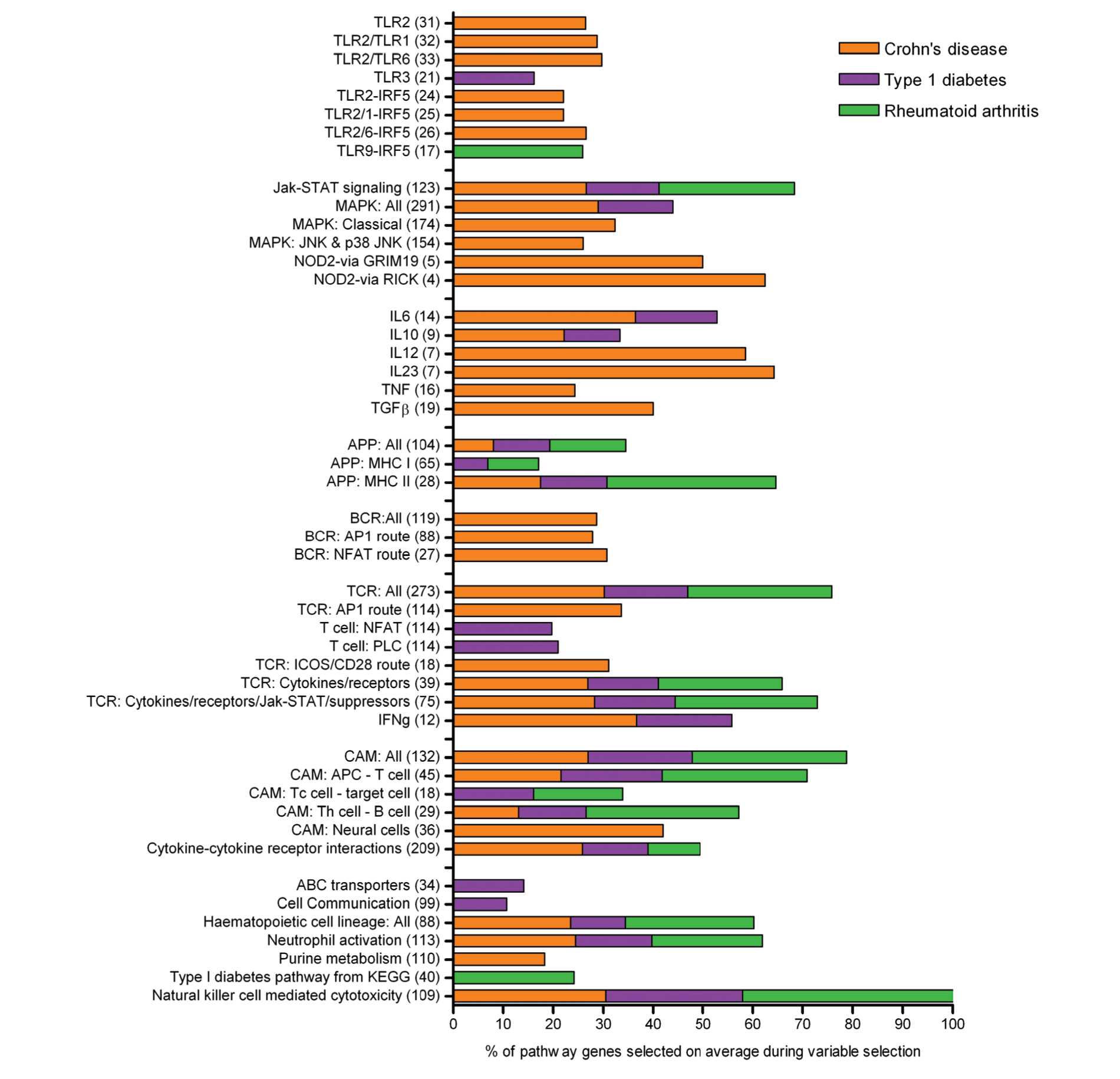

Supplement: Figure S1 — Fraction of the associated pathways selected on average by variable selection. The bar charts show for each disease the average number of genes in a pathway, selected during variable selection, divided by the total number of genes in that pathway and expressed as a percentage. Only associated pathways are shown. The total number of genes in a pathway are shown in parenthesis after the pathway name. The colour-coded bars for each disease are not stacked (i.e. they are not summated). (0.85 MB TIF) [file pone.0008068.s001.tif]

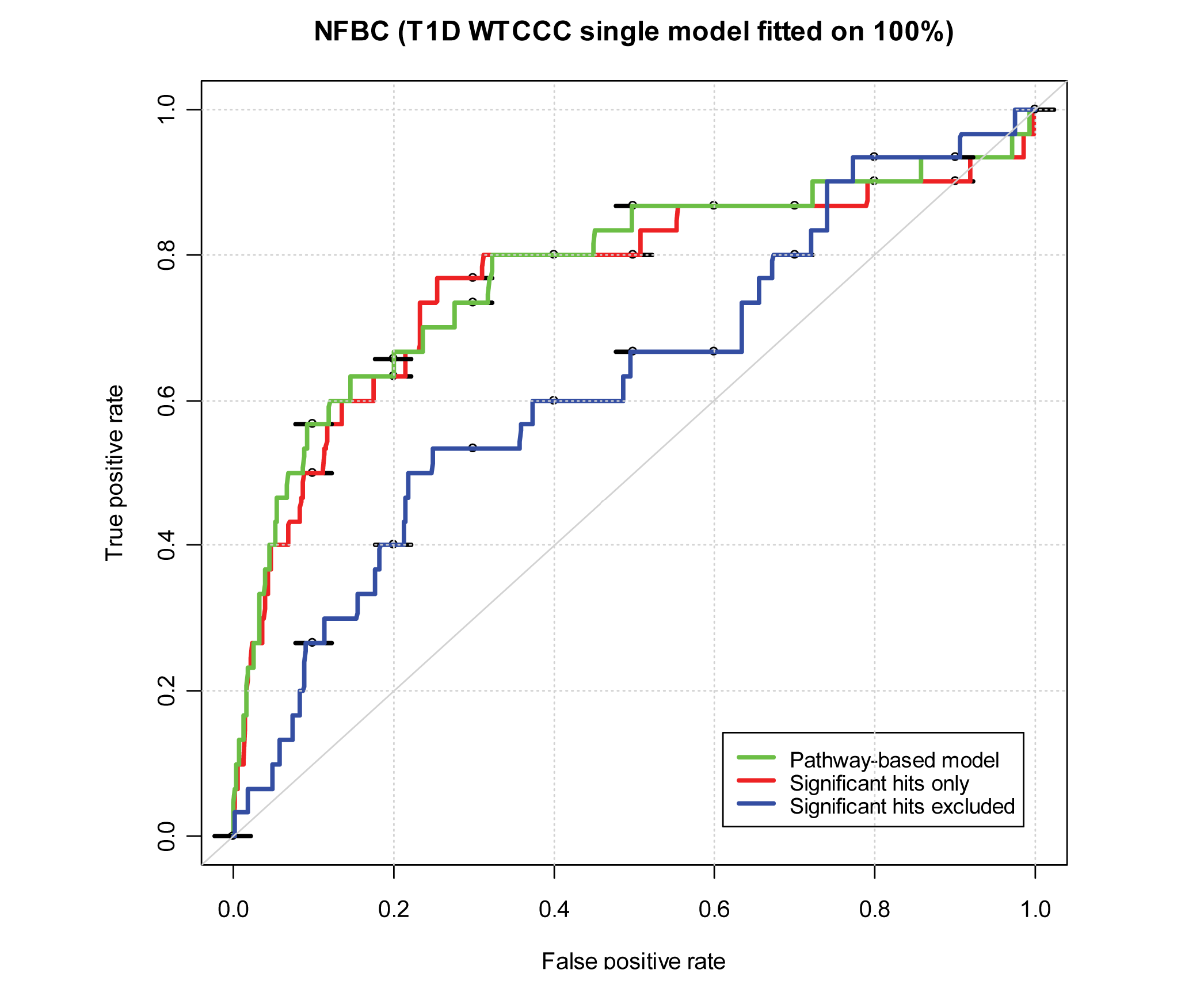

Supplement: Figure S2 — Single model fitted on the entire WTCCC T1D cases and controls when applied to the Northern Finland 1966 Birth Cohort. The area under the ROC curve for the pathway-derived model is 0.77 (green curve), for the same model but with all significant hits (single SNP trend test P<5×10^−7^) and the SNPs in LD (r2≥0.3) excluded is 0.69 (blue curve) and for the model with only the excluded SNPs is 0.74 (red curve). (0.43 MB TIF) [file pone.0008068.s002.tif]

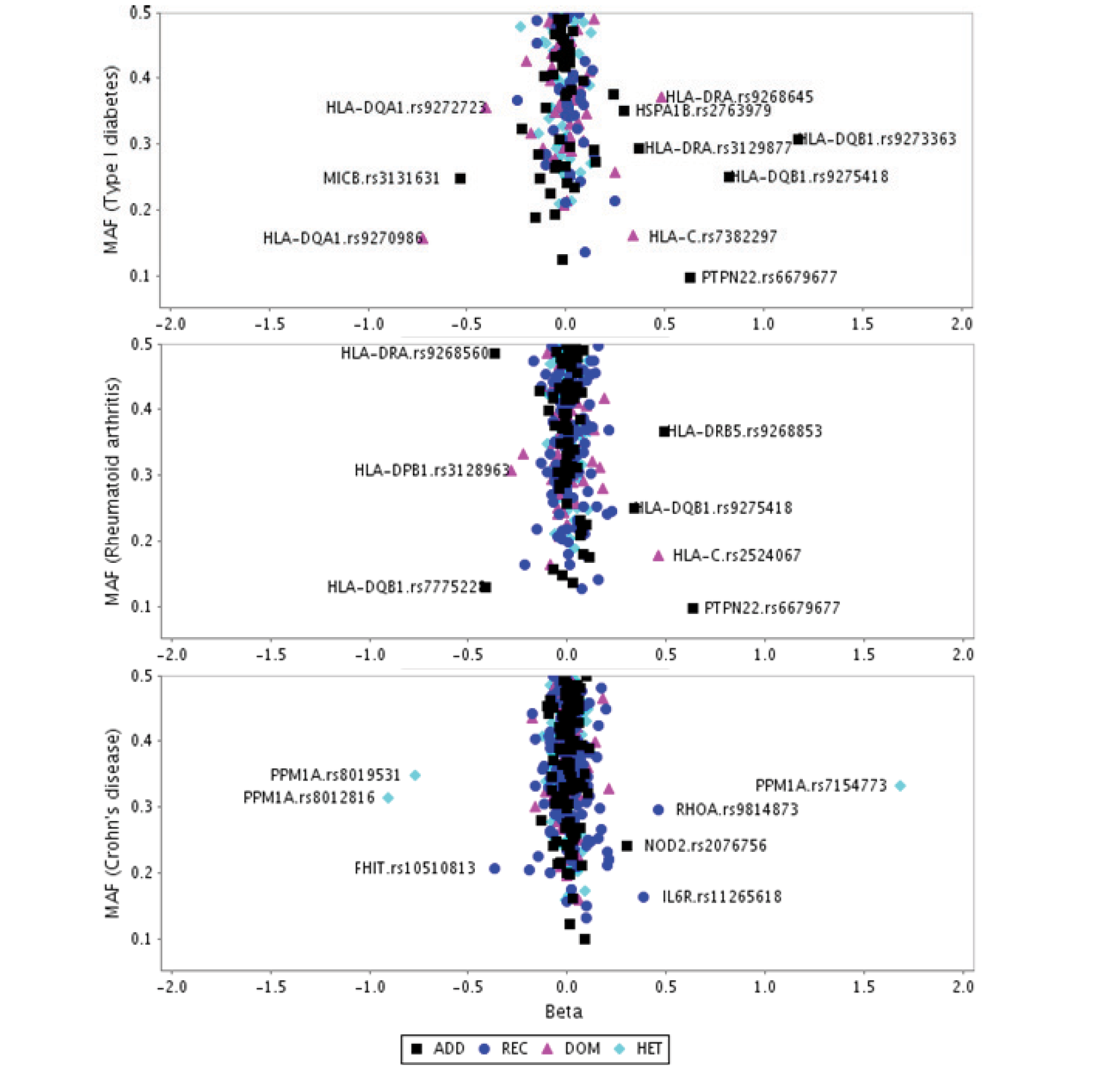

Supplement: Figure S3 — The allelic architecture of the logistic regression models of disease risk. Minor allele frequency vs. beta coefficient for each SNP retained in the fitted logistic regression model from first round of cross-validation. Additive, recessive, dominant and heterozygous effects are displayed by black squares, blue dots, mauve triangles and cyan diamonds respectively. Adverse vs. protective SNPs have a positive vs. negative beta value respectively. Labels are given for all SNPs with beta greater than 0.25. In T1D there are a number of SNPs with large adverse effects acting in a dominant and additive manner and hence a significantly different sum of additive (t-test, P = 2×10e-06) and dominant (P = 2×10e-06) effects between cases and controls. In RA, we observe SNPs with large additive adverse and protective effects resulting in a significant difference in the sum of additive (P = 2×10e-14), as well as dominant (P = 6×10e-04) and recessive (P = 0.014) effects. In CD there are fewer SNPs with large effect yet still significant differences for additive (P = 4e-03) and heterozygous (P = 0.04) cumulative effects. (1.17 MB TIF) [file pone.0008068.s003.tif]

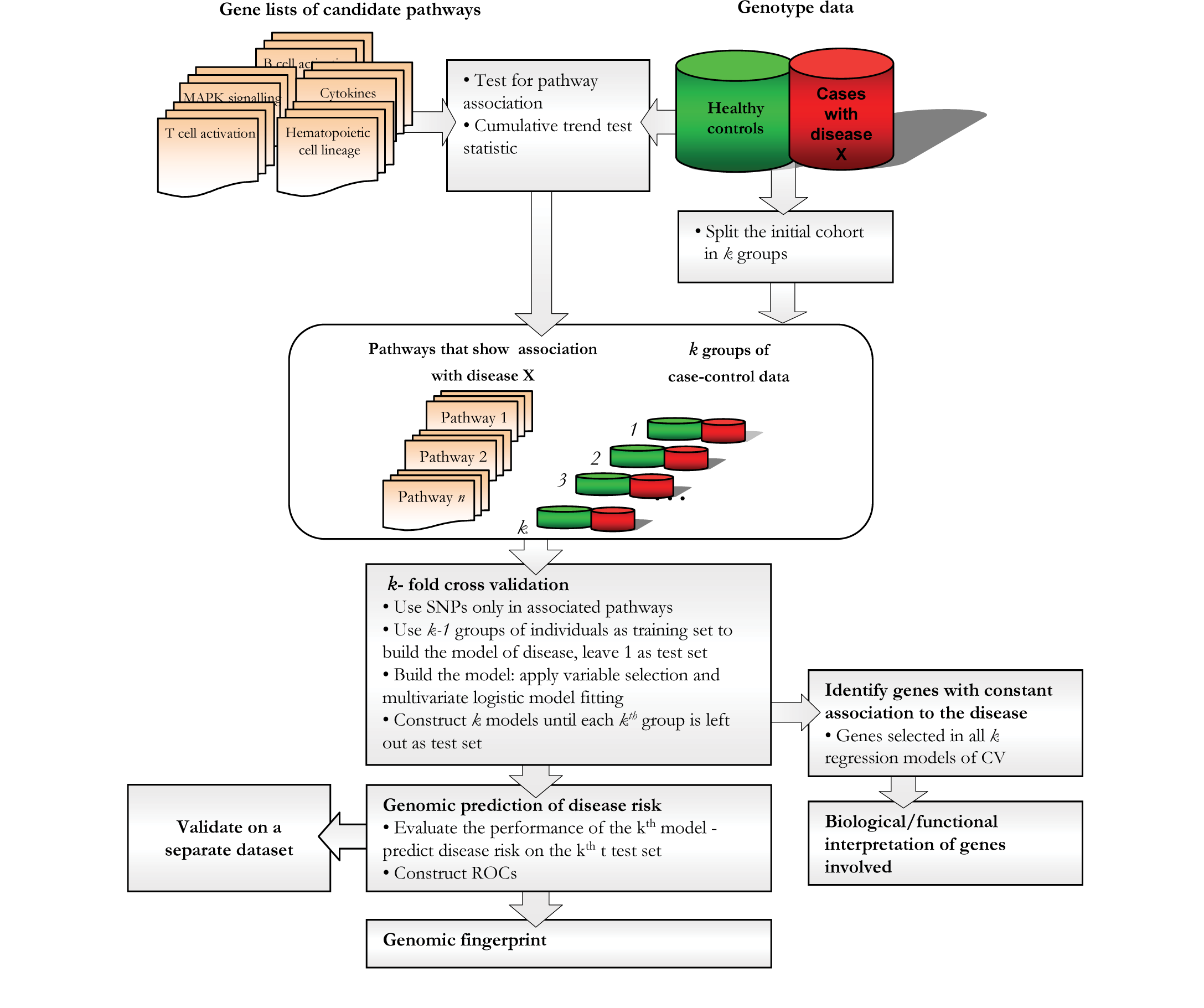

Supplement: Figure S4 — Stepwise procedure for the pathway-based analysis. (0.66 MB TIF) [file pone.0008068.s004.tif]

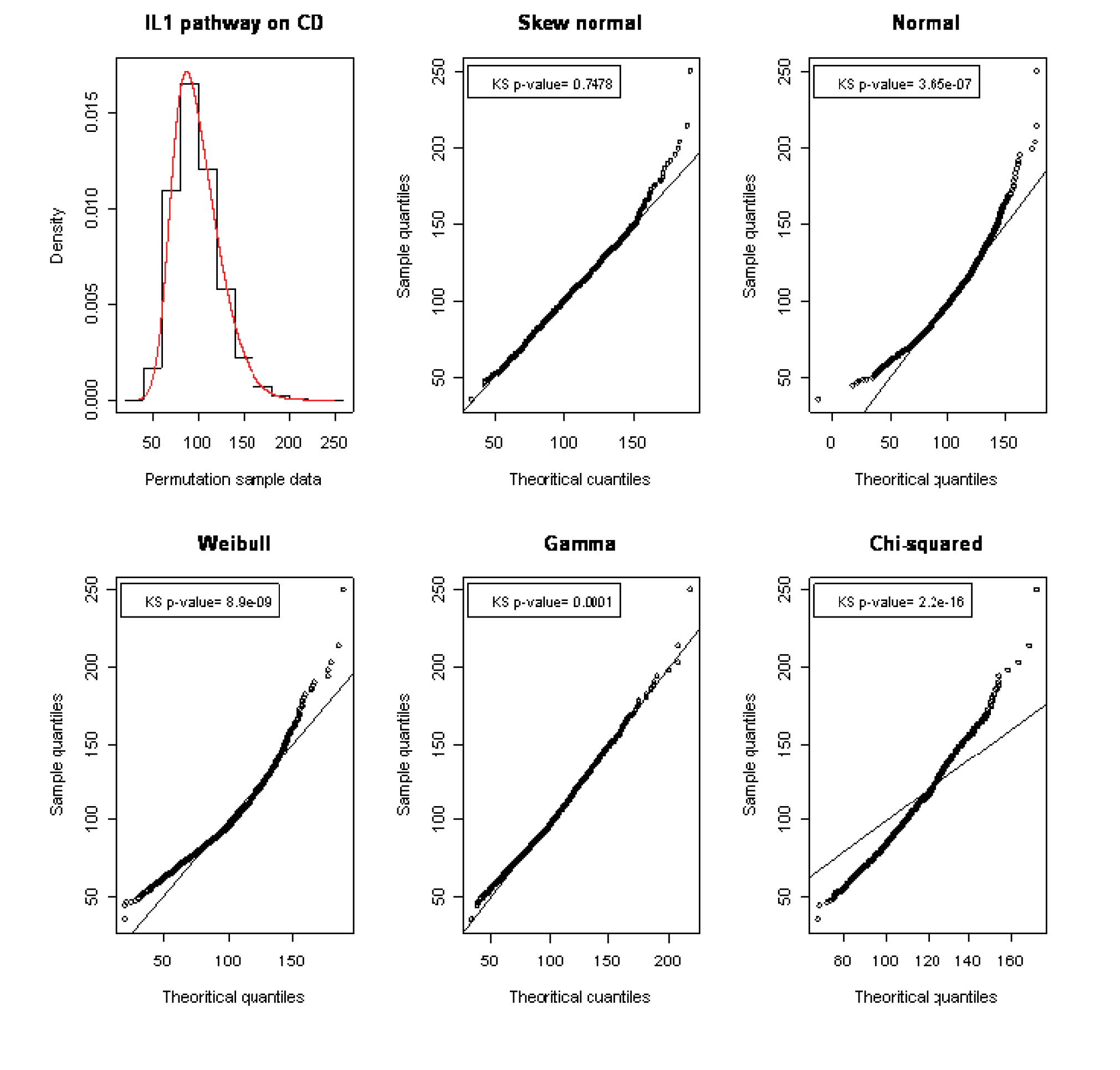

Supplement: Figure S5 — Histogram, density function and q-q plots of various distribution fits to permutation data of the cumulative trend test statistic. The plot on the top left corner shows the histogram and the fitted skew normal density function of the cumulative trend test statistic calculated from 10,000 permutations of cases/control label for the IL-1 pathway in CD and the top middle plot shows the QQ-plot of the fitted skew normal distribution. The next four plots correspond to QQ-plots of four distribution fits to the same null distribution. The P-value of Kolmogorov-Smirnof goodness of fit test statistic is depicted in the legend of each plot. (0.41 MB TIF) [file pone.0008068.s005.tif]
