# Supplementary material for: Pathway Analysis of GWAS Provides New Insights into Genetic Susceptibility to 3 Inflammatory Diseases
Source: PLoS One. 2009 Nov 30;4(11):e8068. doi: 10.1371/journal.pone.0008068 (PMC2778995; doi:10.1371/journal.pone.0008068)
Supplement: Methods S1 — (11.21 MB PDF) [file pone.0008068.s010.pdf]

Supplementary Methods 1

Patients and controls. We analysed the raw genotypic data from the Wellcome Trust Case Control Cohort study on 14,000 Caucasian UK patients and 3000 controls genotyped on the Affymetrix 500K mapping array GWA GeneChip. The cohort included 7 common diseases; Crohn's disease, rheumatoid arthritis, type 1 diabetes, hypertension, type 2 diabetes, bipolar disorder and coronary artery disease. The full clinical details, genotype data and initial analyses are described in Nature 2007 Jun 7; 447(7145): 661-78. In the original work, 16,179 individuals remained in the study after filtering for contamination, false identity, non-Caucasian ancestry and relatedness, while 468,919 SNPs passed all the quality control filters and the visual inspection of the genotype call cluster plots. We followed the same approach and used this dataset in our study.

For the validation study we used the National Finland 1996 Birth Cohort. The cohort followed 12,058 infants, born in 1966 for over 30 years, with documentation of all health events, and illnesses and genotyped using the Illumina Infinium 370cnvDuo array[1]. Over the 30 years of follow up, 30 of the 4,763 genotyped individuals were diagnosed as having T1D, and were the cases in the validation study.

Pathway selection. The pathways under investigation were defined using published annotation from the KEGG[2] database and Ingenuity Pathways Analysis 6, supplemented from the literature. All examined pathways and the genes that comprise each are listed in tables on the web information (submitted for review). Gene to SNP mapping was performed from the Affymetrix annotation files. We used all SNPs within 10KB from each side of the gene. We examined 84 pathways and sub-pathway

combinations in total, where 1368 genes are involved and 37495 SNPs are assigned to them. All genes are referred to by their HGNC ID and SNPs by their dbSNP id.

Test for pathway association- cumulative trend test statistic. To obtain the cumulative trend test statistic for pathway association, we summed the Armitage trend test statistic over all of the *n* SNPs in a given pathway. If we denote as X the Armitage trend test statistic for the ith SNP, then the cumulative trend test statistic for the pathway is $CT_{pathway} = \sum_{i=1}^{n} X_i$, where *n* is the number of SNPs in the pathway. We estimated the parameters of a parametric approximation of the null distribution of the statistic by fitting a skew normal distribution to results obtained from 1000 random permutations of case/control label. For each disease and pathway we chose location (μ), scale (σ) and shape (λ) parameters which maximise the likelihood $P(CT_j \mid \mu, \sigma, \lambda), j \in [1,1000]$, where each CT_j is calculated as the cumulative statistic under a random permutation of the case control labels and *j* is the index of a random permutation. We then estimated the significance as $P = \int_{CT_{pathway}}^{\infty} P(x \mid \mu, \sigma, \lambda) dx$, where $CT_{pathway}$ is the pathway statistic under no permutation and $P(x \mid \mu, \sigma, \lambda)$ is the fitted skew normal distribution. This procedure was carried out separately for each disease and given pathway using the SN package in R[3]. To estimate a parametric approximation of the null distribution of the statistic we fitted several continuous theoretical distributions. The skew-normal approximation was finally preferred as it exhibited better fit that the Normal, Weibull and Gamma distributions (Supplementary Figure 5). Goodness of fit was assessed by QQ-plots of the observed and theoretically distributed data and subsequently by the Kolmorogov-Smirnof goodness of fit test statistic.

The *P*-values from the cumulative trend test statistic reported in Table 1 were evaluated on all individuals. However, we also calculated the cumulative trend test statistic on the same 90% used in the first round of cross validation, and found that the same pathways (with the exception of MAPK in T1D and TLR2-TLR1 signalling via IRF5, T cell activation via NFAT, and cytotoxic T cell to target cell signalling in CD) were identified as significant in all three diseases, but at a higher threshold of 0,004. None of the SNPs in the HyperLasso model for this round of cross-validation were unique to these excluded pathways.

We also examined the extent to which SNPs significant on their own effects drove the association of a pathway as a whole. We repeated the cumulative trend test statistic calculation for each pathway without the significant hits and those in LD with them. Results are shown in Supplementary Table 4.

Variable selection and logistic regression. Variable selection and model fitting was performed with the program HyperLasso[4] which is implemented in C. No interactions were taken into account. The algorithm fits a logistic regression model while performing variable selection to generate models with relatively few genotype predictors. HyperLasso was applied as to consider four types of SNP effects - additive, recessive, dominant, heterozygote – and encoded genotypes accordingly – [0,1,2], [0,0,x], [0,y,y], [0,z,0], where the original genotypes are ordered as [AA, Aa, aa] and a is the minor allele. The values of x, y and z are arbitrary and affect the probability of each effect type being included in the model; we chose the values of x, y and z for each SNP separately such that the standard deviation over all SNPs of all four effect types was the same. This resulted in choosing the effect type with the largest gradient of the log-likelihood at each SNP, which is equivalent to choosing the

effect type with the minimum *P* value (see Supplementary Methods 2). The algorithm runs with two parameters which affect the Normal Exponential Gamma (NEG) prior used on the coefficients in the logistic regression model. The first is the shape parameter, which we set to 3.5, and the second is the derivative of the log of the density function at the origin which we set to 40.

Model evaluation and ROCs. To evaluate the performance of the predictive logistic models, we performed 10-fold cross-validation. As in the general case of k- fold cross-validation, the dataset was divided into k subsets (in this study k=10) and each time, one of the k subsets was used as the test set and the other k-1 subsets were considered together to form a training set. Variable selection and model fitting was performed on each kth training dataset separately and the predictive power of the kth fitted logistic model was evaluated on the corresponding k^{th} test set. The average sensitivity/specificity across all *k* trials was computed to produce the average ROC plot (Figure 3). The area under the ROC curves (AUC) was calculated following the process outlined in Mason and Graham (2002)[5].

The procedure was repeated for each disease. Specifically, the initial cohorts of T1D, RA and CD (1963, 1860 and 1748 cases), were split into 10 datasets each of 196, 186 and 174 individuals respectively. The control group of 2938 individuals was split into subsets of 293 individuals. During each trial, a control subset with each disease specific subset formed the *test* dataset for the particular disease, while the 9 remaining disease and controls subsets were put together to form the *training* set. For the validation study, we took the 10 models fitted during cross-validation on the WTCCC, as well as a single model fitted on 100% of the WTCCC data, and evaluated each of these models in the NFBC. All non-genotyped SNPs were imputed using IMPUTE[6]

to infer missing genotypes at Affymetrix 500K probe positions in the NFBC dataset, using a CEPH Hapmap[7]. The imputed genotype with the highest probability was assigned to each individual. We evaluated the predictive power with ROC curves. The results are shown on Figure 3D.

***Adverse* and *protective* genotype analysis.** For each disease, we picked the logistic model trained on the first fold of the 10 fold CV to demonstrate the *adverse* against *protective* genotype effect. We repeat the same procedure for all models and the plots remain the same (data not shown). We defined each SNP as either *adverse* or *protective* according to the sign of the coefficient (+ve or –ve) in the logistic model relative to the genotypic encoding at this SNP (see Variable selection). For every individual we calculated the counts of *adverse* and the counts of *protective* effects as well as their ratio.

Graphical display of adverse and protective genotypes in each individual. We displayed all cases and controls in relation to the variants carried by each individual. Genotypes were coded by the variable selection algorithm coding scheme. Cells were calculated as the product of the coefficients of the logistic model with each individual's genotypes. Thus, for cell $c(i, j)$ it would be $c(i, j) = \beta(i) x gen(i, j)$ where $\beta(i)$ is the logistic model coefficient at SNP i and $gen(i, j)$ is the genotype of individual j at SNP i. Cells were coded red at *adverse* SNPS, and green at *protective* SNPS. The intensity of the colour in each cell was calculated using the cumulative distribution function (cdf) of the normal distribution as $intensity = 2 * (cdf(abs(c(i, j)) \mid \mu = 0, \sigma) - 0.5)$, where σ is calculated as sigma = $\sigma = \sqrt{\sum_j c(i, j)^2}$. SNPs were ordered via a neighbour-joining based tree-clustering

algorithm designed to place rows with similar patterns next to each other. To do this we first calculated a distance between each SNP i and k as $d(i,k)=\sum_j (c(i,j)-c(k,j))^2$, and then calculated a neighbour joining tree from this distance matrix in a manner similar to Howe et al (2002)[8].

References

1. Sabatti C, Service SK, Hartikainen AL, Pouta A, Ripatti S, et al. (2009) Genome-wide association analysis of metabolic traits in a birth cohort from a founder population. Nat Genet 41: 35-46.
2. Kanehisa M, Araki M, Goto S, Hattori M, Hirakawa M, et al. (2008) KEGG for linking genomes to life and the environment. Nucleic Acids Res 36: D480-484.
3. Azzalini A, Capitanio A (1999) Statistical applications of the multivariate skew-normal distribution. Journal of the Royal Statistical Society 61: 579-602.
4. Hoggart CJ, Whittaker JC, De Iorio M, Balding DJ (2008) Simultaneous analysis of all SNPs in genome-wide and re-sequencing association studies. PLoS Genet 4: e1000130.
5. Mason SJ, Graham NE (2002) Areas beneath the relative operating characteristics (ROC) and relative operating levels (ROL) curves: Statistical significance and interpretation. Q J R Meteorol Soc 128: 2145–2166.
6. Marchini J, Howie B, Myers S, McVean G, Donnelly P (2007) A new multipoint method for genome-wide association studies by imputation of genotypes. Nat Genet 39: 906-913.
7. Manolio TA, Brooks LD, Collins FS (2008) A HapMap harvest of insights into the genetics of common disease. J Clin Invest 118: 1590-1605.
8. Howe K, Bateman A, Durbin R (2002) QuickTree: building huge Neighbour-Joining trees of protein sequences. Bioinformatics 18: 1546-1547.

Supplementary Methods 2

Variable selection and logistic regression using shrinkage priors

Variable selection and model fitting was implemented simultaneously in the program HyperLasso[1] . The algorithm fits a logistic regression model, simultaneously performing variable selection to generate models with relatively few predictors - SNPs. The regression coefficients are given by maximum posterior estimates and sparse solutions are achieved by using a prior with a sharp peak at zero. The prior used was the normal-exponential-gamma (NEG) distribution which is a generalisation of the commonly used double-exponential (DE) prior. Analytic representation of the distribution is given in [1]. The NEG is characterised by two parameters, a shape and a scale parameter, whereas the DE is characterised by a single parameter. The DE is a special case of the NEG, but otherwise the NEG has a sharper peak at zero and heavier tails than the DE.

Posterior modes are found by updating each regression coefficient one at a time using optimisation steps similar to Newton's method. The algorithm cycles through the covariates until a convergence criterion is met; see [1] and [2], for details of the update steps and convergence criterion. The algorithm searches for four effect types: additive, dominant, recessive and heterozygous. When a SNP is not currently included in the model (its regression coefficient equals zero), the four effect types are all considered, and only the one with the largest gradient of the log-likelihood at the origin is considered for entry into the model. Once included in the model, only the parameter value is updated at subsequent visits of the algorithm to this SNP, and not the effect type; the latter can only change within an iteration by the parameter value reverting to zero and a different model being selected in subsequent cycles.

In [1] the genotype data was standardised such that all markers had standard deviation one. This is equivalent to specifying a prior distribution that favours larger effect sizes at SNPs with lower MAF and gives a procedure that is equivalent to the Armitage Trend Test when applied univariately. The analyses in this paper did not standardise the genotype data, and thus specified the same prior on effect size at all SNPs resulting in an algorithm that

chooses those SNPs which explain the greatest variation in the phenotype. In all analyses the genotype data was centred to have mean zero.

The effect types are coded as: additive, genotypes coded 0,1,2; recessive, genotypes coded 0,0,x; dominant, genotypes coded 0,y,y; heterozygous, genotypes coded 0,z,0, in all cases the first genotype is the homozygote major allele. The values of x, y and z are arbitrary and effect the probability of each effect type being included in the model. We choose the values of x, y and z for each SNP separately such that standard deviation of the all four effect types were the same. This has the effect that our procedure, which chooses the effect type with the largest gradient of the log-likelihood at each SNP, is equivalent to choosing the effect type with the minimum p-value. This is shown below.

The shrinkage algorithm is such that if $\beta = 0$, it will remian at the origin if [1]

$$|L'(\beta = 0)| < f'(\beta = 0) \tag{1}$$

where L' is the derivative of the log-likelihood and f' is minus derivative of the log prior.

The log–likelihood and its first and second derivatives are given by

$$L(\beta) \equiv \log p(\boldsymbol{y}, \boldsymbol{x} \mid \beta) = -\sum_{i=1}^{n} \log\left(1 + \exp\{-\eta_i\}\right)$$

where $\eta_i = y_i(\beta_0 + \beta x_i)$ and β_0 is the intercept.

$$\begin{aligned} L'(\beta) &\equiv \frac{d}{d\beta} L(\beta) = \sum_{i=1}^{n} \frac{x_{ij} y_i}{1 + \exp \eta_i} \\ L''(\beta) &\equiv \frac{d^2}{d\beta^2} L(\beta) = -\sum_{i=1}^{n} x_{ij}^2 \frac{\exp \eta_i}{(1 + \exp \eta_i)^2} \end{aligned} \tag{2}$$

We approximate the log-likelihood using a Taylor series expansion:

$$L(\beta) \approx L(\hat{\beta}) - \frac{1}{2} L''(\hat{\beta} - \beta)^2$$

where $\hat{\beta}$ is the mle. The derivative can be approximated by

$$L'(\beta) \approx -L''(\hat{\beta} - \beta)$$

Evaluating at $\beta = 0$,

$$L'(\beta) \approx -\hat{\beta} L''(\hat{\beta})$$
$$= \hat{\beta} \sum_{i=1}^{n} x_i^2 \frac{\exp \eta_i}{(1 + \exp \eta_i)^2}$$

For small $\hat{\beta}$

$$\approx \hat{\beta} \frac{\exp\{\beta_0\}}{(1 + \exp(\beta_0))^2} \sum_{i=1}^{n} x_i^2$$

Thus the criteria in (1) can be approximated by

$$\hat{\beta} < \frac{\kappa}{\sum_{i=1}^{n} x_i^2} \tag{3}$$

Under the null the distribution of $\hat{\beta}$ is [3]

$$\hat{\beta} \sim \mathrm{N}\left(0, -L''(\beta = 0)\right)$$
$$\sim \mathrm{N}\left(0, \frac{\exp\{\beta_0\}}{(1 + \exp(\beta_0))^2} \sum_{i=1}^{n} x_i^2\right),$$

Thus if $\sum_{i=1}^{n} x_i^2$ is set to be equal for all effect types the distribution of $\hat{\beta}$ will be the same for all effect types and thus from (3) all effect types will have equal probability of entering under the null.

This procedure is equivalent to choosing the effect type with the minimum p-value as we show below. The Armitage trend test is equivalent to a score test and thus can be espressed as

$$T = \frac{L'(\beta = 0)}{\sqrt{-L''(\beta = 0)}}$$

Applying the Taylor series approximations for L gives

$$\approx \hat{\beta} \sqrt{\frac{\exp\{\beta_0\}}{(1 + \exp(\beta_0))^2} \sum_{i=1}^{n} x_i^2}$$

Thus the criteria for the null $\beta = 0$ to be accepted can be expressed as

$$\hat{\beta} < \frac{\kappa'}{\sqrt{\sum_{i=1}^{n} x_i^2}}$$

Since κ' is the same for all effect types (function of intercept and significance threshold) and $\sum_{i=1}^{n} x_i^2$ has been set to be equal for all effect types our procedure is equivalent to choosing the effect type with the minimum p-value.

References

[1] Hoggart CJ, Whittaker J, De Iorio M, Balding DJ Simultaneous analysis of all snps in genome-wide and re-sequencing association studies. Plos Genetics 4.

[2] Genkin A, Lewis DD, Madigan D (2007) Large-scale Bayesian logistic regression for text categorization. Technometrics 49(3):291–304.

[3] Cox DR, Hinkley DV (1974) Theoretical statistics. London: Chapman and Hall.
